# 2β-(Isobutyryloxy)florilenalin, a Sesquiterpene Lactone Isolated from the Medicinal Plant *Centipeda minima*, Induces Apoptosis in Human Nasopharyngeal Carcinoma CNE Cells

**DOI:** 10.3390/molecules14062135

**Published:** 2009-06-12

**Authors:** Miaoxian Su, Yaolan Li, Hau Yin Chung, Wencai Ye

**Affiliations:** 1 Department of Biology, The Chinese University of Hong Kong, Hong Kong, China; E-mail: sumiaoxian@yahoo.com.cn (M-X.S.); 2 Institute of Traditional Chinese Medicine & Natural Products, College of Pharmacy, Jinan University, Guangzhou 510632, China; E-mail: tliyl@jnu.edu.cn (Y-L.L.); 3 Guangdong Province Key Laboratory of Pharmacodynamic Constituents of TCM and New Drug Research, Guangzhou 510632, China; E-mail: chywc@yahoo.com.cn (W-C.Y.); 4 Food and Nutritional Sciences Programme, The Chinese University of Hong Kong, Hong Kong, China

**Keywords:** 2β-(isobutyryloxy)florilenalin, *centipeda minima*, sesquiterpene lactone, apoptosis, nasopharyngeal carcinoma

## Abstract

*Centipeda minima* is a medicinal plant reputed in China as a remedy for nasopharyngeal carcinoma (NPC). In this study, bioactivity-guided fractionation of the anti-NPC compound(s) from *C. minima* led to the isolation of 2β-(isobutyryloxy)florilenalin (IF), a sesquiterpene lactone. IF showed significant dose- and time- dependent inhibition on the growth of the human nasopharyngeal carcinoma epithelia cells (CNE). It induced apoptosis in CNE cells, as shown by the accumulation of sub-G1 cell population, DNA fragmentation and nuclear condensation, caspase-3 activation and PARP cleavage. Such induction was associated with the depletion of mitochondrial membrane potential (ΔΨm) and the release of cytochrome *c* to cytosol to regulate the expression of Bcl-2 family proteins. These activities led to the cleavage of caspases and the trigger of cell death process. Overall, IF in *C. minima* showed potent antiproliferative effect of *C. minima* on NPC cells, suggesting that the plant deserves more extensive investigation for its potential medicinal application.

## 1. Introduction

*Centipeda minima* (L.) A. Br. (Compositae) is an annual plant distributed throughout high humidity geographic locations throughout China, Korea, Japan, India, Malaysia and Oceania [1]. Six *Centipeda* species are known, but only *C. minima* grows in China [1]. The whole plant, which is harvested during its anthesis in both summer and autumn, has pharmaceutical applications [2]. Based on the record in the Pharmacopoeia of China [2], the dried herb is used to treat nasal allergies, rhinitis and sinusitis, coughs and headaches. In addition, it is used in the Chinese folk medicine to treat nasopharyngeal carcinoma (NPC) [3,4]. Recent pharmacological interest has focused on its anti-allergy and anti-bacterial effects [5,6,7,8]. Phytochemical studies of its composition have led to the identification of a number of terpenes, including sesquiterpene lactones and triterpenes [5,6,9]. The former class contained the major active constituents contributing to the anti-allergy and anti-bacterial activities of the herb. Despite increasing evidence demonstrates the anticancer potential of sesquiterpene lactones [10], there is a paucity of information [11] on the anticancer activities of sesquiterpene lactones in *C. minima*. Besides, both the anti-nasopharyngeal carcinoma potential and the potent constituents of *C. minima* remain elusive.

The objectives of this study were to isolate and identify the active principal(s) in *C. minima* based on bioactivity-guided fractionation using human nasopharyngeal carcinoma epithelia cells (CNE) as the target and elucidate the underlying mechanism leading to the cell death.

## 2. Results and Discussion

**Scheme 1:**
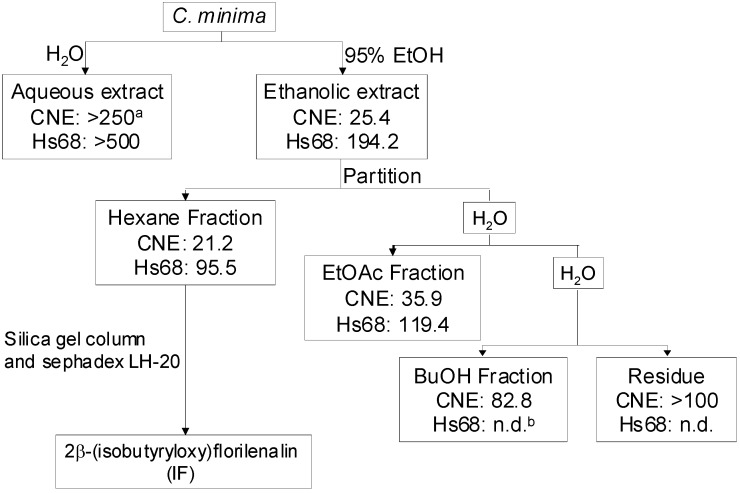
Summary of the bioactivity-guided isolation of *Centipeda minima* by using CNE (human nasopharyngeal cancer cells) and Hs68 (human normal skin cells) cells at 72-h treatment. ^a^ Inhibition concentration (IC_50_) value in μg/mL. ^b^ not determined.

In this study, an MTT assay, which is based on the ability of a mitochondrial dehydrogenase enzyme from viable cells to cleave the tetrazolium rings of the pale yellow MTT and form a dark blue formazan crystals, was employed to screen the inhibitory effects of both aqueous and ethanolic extracts from *C. minima* on CNE cells. Results showed that CNE cells were more susceptible to the ethanolic extract than to the aqueous one (Scheme 1). The IC_50_ value of the ethanolic extract was 25.4 µg/mL, whereas that of the aqueous one was much larger (>250 µg/mL). As the crude ethanolic extract was more potent, it was further fractionated with organic solvents of different polarities. The inhibitory effects of the fractions on the CNE cells increased as polarity of the solvents decreased and the hexane fraction, having the lowest polarity, was found to be the most potent. The corresponding IC_50_ values of the hexane, EtOAc, butanol fractions and the residue on the CNE cells were found to be 21.2, 35.9, 82.8 and >100 µg/mL, respectively. Normal human skin Hs68 cells were weakly affected by the extracts and fractions. 

**Figure 1:**
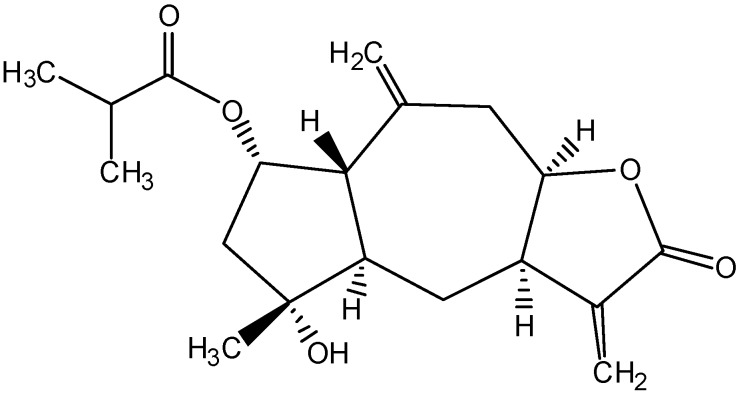
Chemical structure of IF isolated from *Centipeda minima*.

As the hexane fraction was the most effective, it was subjected to further fractionation to search for the active principal(s). This led to the purification of 2β-(isobutyryloxy)-florilenalin (IF), a sesquiterpene lactone (Figure 1), and its positive identification by spectroscopic methods and comparison with the published data in literature [9]. 

Sesquiterpene lactones, most widely distributed within the *Compositae*, have received considerable attention for their anticancer properties [10]. The highly electrophilic α,β-unsaturated carbonyl structures, such as the α-methylene-γ-lactone ring and the α,β-unsaturated cyclopentenone, are considered as the general bioactive functional groups in sesquiterpene lactones [10], as they allow the structures to interact rapidly with the nucleophilic sites of biological molecules in a Michael-type addition. Covalent binding of sesquiterpene lactones to free sulfhydryl groups in proteins is possible and may interfere with the normal protein function. Besides, alkylation with the DNA molecules presents a potential molecular cytotoxicity for cells [12]. Previous phytochemical studies of *C. minima* have identified more than ten sesquiterpene lactones which are all pseudo-guanianolide or guaianolide types [5,6,7,9,13]. Among them, 6-*O*-angeloylenolin, a sesquiterpene lactone containing an α,β-unsaturated cyclopentenone, isolated from *C. minima* was reported to induce apoptosis in HL-60 cells *in vitro* and inhibit the solid cancer growth in Lewis lung cancer xenograft model [11]. IF, isolated in this study, contains a bioactive functional group of sesquiterpene lactone, the α-methylene-γ-lactone ring. This led to our deduction that IF might possess similar apoptotic activity. However, other than a report on the sensitivity Gram-positive bacteria [8], no other information has reported such activity in IF.

**Figure 2:**
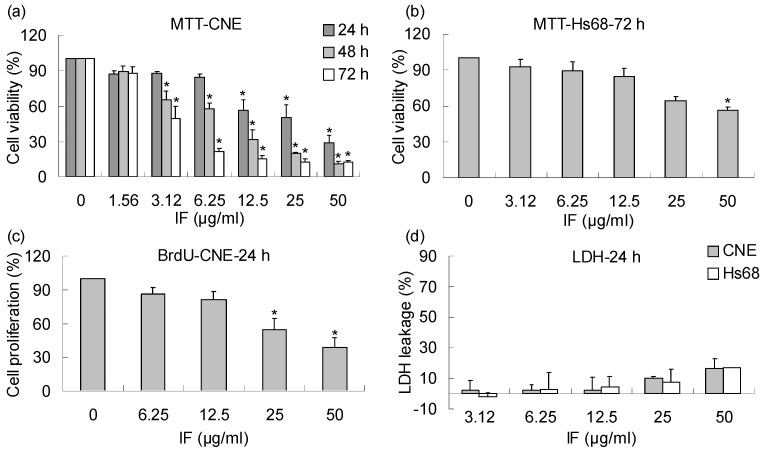
Effects of IF on the growth of CNE and Hs68 cells. (a) Effect of IF on the viability of CNE cells at 24-, 48-, 72-h treatments evaluated by MTT assay. (b) Effect of IF on the viability of Hs68 normal cells at 72-h treatment evaluated by MTT assay. (c) Effect of IF on the proliferation of CNE cells at 24-h treatment evaluated by BrdU assay. (d) Cytotoxic effects of IF on CNE and Hs68 cells at 24-h treatment evaluated by LDH assay. Each value represents the mean of three independent experiments. Difference between treatments and control with p < 0.05 (*) was considered statistically significant.

In the present study, the MTT results showed that IF exhibited significant dose- and time- dependent effects on the growth of CNE cells, with IC_50_ values of 25.6 (24 h), 8.1 (48 h) and 3.1 μg/mL (72 h), respectively (Figure 2a). Despite its potency in CNE cells, IF exerted lower inhibitory effect on the normal Hs68 cells with an IC_50_ value larger than 50 μg/mL at 72-h treatment (Figure 2b). Similar dose-dependent inhibitory effect was observed in the ELISA-BrdU assay for 24-h incubation, which is based on the incorporation of the pyrimidine analogue BrdU instead of thymidine into the DNA of proliferating cells (Figure 2c). Lactate dehydrogenase (LDH) is a stable cytoplasmic enzyme present in cells and rapidly released into the cell culture supernatant upon damage of the plasma membrane. Hence, the activity of LDH correlates to the number of lysed cells. In this study, no significant cytotoxic effects on both CNE and Hs68 cells were found when the leakage of LDH into the cell medium was evaluated (Figure 2d).

**Figure 3:**
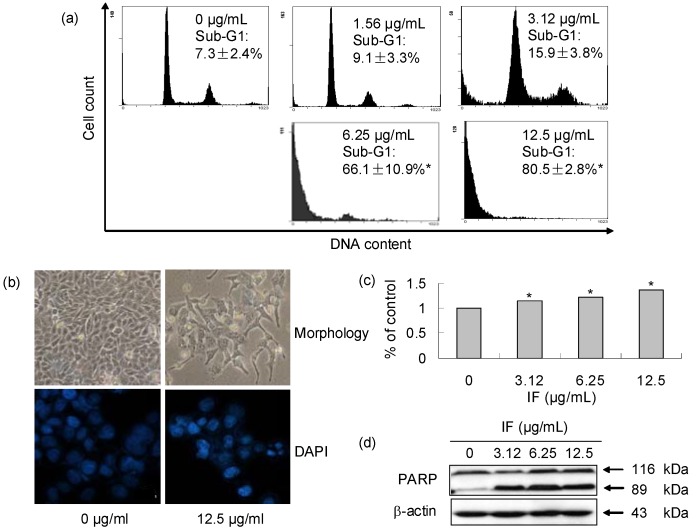
Apoptosis induction of IF in CNE cells at 72-h treatment. (a) Effect of IF on cell cycle distribution analyzed by flow cytometry. (b) Effect of IF on the morphology of CNE cells. Morphology (upper): phase-contrast microscopy. DAPI (lower): fluorescence microscopy. (c) Activation of caspase by IF. (d) IF induced PARP cleavage. Each value represents the mean of three independent experiments. Difference between treatments and control with p < 0.05 (*) was considered statistically significant.

**Figure 4:**
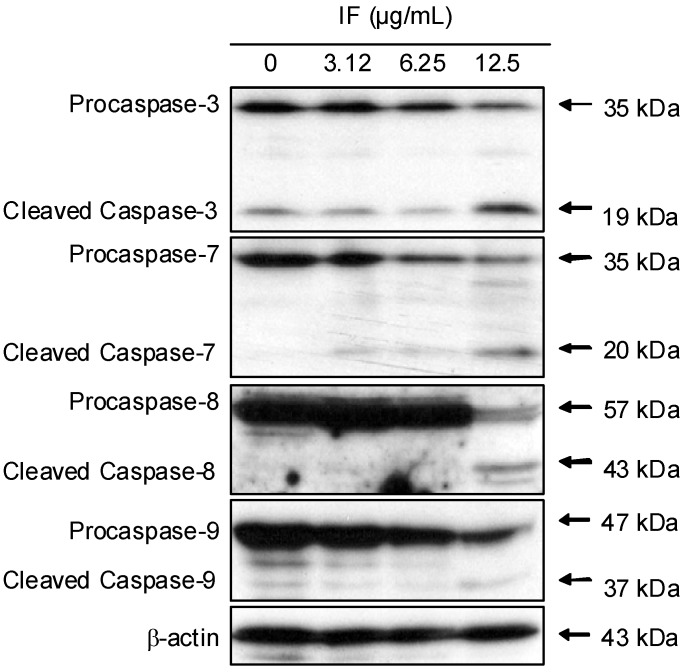
Effect of IF on the expression of caspase-3, -7, -8, and -9 in CNE cells at 72-h treatment. β-actin was used as the loading control. Results were representatives of three independent experiments.

As IF demonstrated strong antiproliferative effect on the CNE cells, its underlying mode of action was investigated. Apoptotic cells, which have a decreased content of DNA when compared to living cells, will have a hypo-diploid DNA content (sub-G1 phase) when analyzed in a flow cytometer after staining with propidium iodide (PI). Therefore, DNA cell cycle analysis of CNE cells treated with IF was carried out. Results showed that the accumulation of cells in the sub-G1 phase significantly increased in a dose-dependent way (Figure 4a), suggesting apoptosis was induced by IF. The sub-G1 cell populations were 7.3 ± 2.4, 9.1 ± 3.3, 15.9 ± 3.8, 66.1 ± 10.9 and 80.5 ± 2.8% with IF concentrations of 0, 1.56, 3.12, 6.25 and 12.5 µg/mL, respectively. Additional confirmations of apoptosis in the presence of IF were from the measurements of three other apoptotic markers including DNA fragmentation, caspase-3 activation, and PARP cleavage (Figure 3b, Figure 3c, and Figure 3d). Irregularity in shape and cellular detachment in the treated CNE cells (12.5 μg/mL of IF) were observed under a phase-contrast microscopy (Figure 3b). Similarly, DNA fragmentation and nuclear condensation were observed in the treated CNE cells (12.5 μg/mL of IF) after DAPI staining. Our data also revealed that IF induced significant dose-dependent increase in caspase-3 activity in CNE cells after 72-h treatment (Figure 3c). Additionally, cleavage of PARP with treated cells undergoing apoptosis resulted in the presence of an 89 kDa fragment (Figure 3d). These results proved strongly that IF induced apoptosis in CNE cells. 

**Figure 5:**
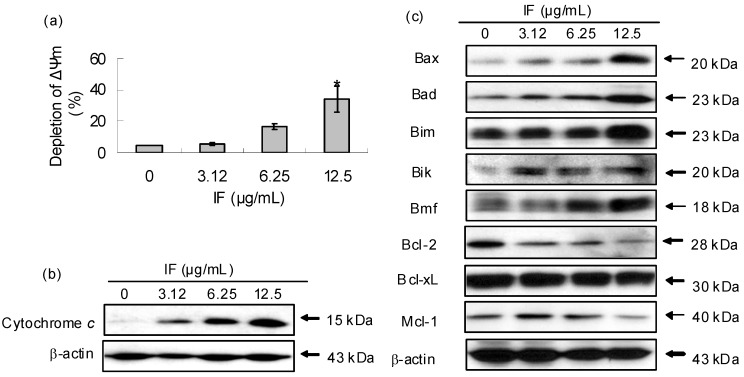
Effect of IF on mitochondria and the related protein expression in CNE cells at 72-h treatment. (a) Effect of IF on mitochondrial membrane potential (ΔΨm). Each value represents the mean of three independent experiments. Difference between treatments and control with p < 0.05 (*) was considered statistically significant. (b) The level of cytochrome *c* in the cytosol. (c) Effect of IF on the expression of Bcl-2 family proteins. β-actin was used as the loading control. Results were representatives of three independent experiments.

Caspases are a family of cystein-dependent asparate-directed proteases that act as important regulators in apoptosis cascade. They are important components in the two apoptotic pathways, namely, the extrinsic and the intrinsic pathways [14]. Similar to the proteolytic cleavage of PARP, the two downstream effectors, namely caspase-3 and -7, were cleaved to become the active forms (Figure 5). Caspase-8 is regarded as the key initiator in the extrinsic pathway. Figure 4 shows that the level of procaspase-8 was significantly reduced at the concentration of 12.5 μg/mL, whereas the cleaved product, caspase-8, was also found. Caspase-9 is the central initiator in the intrinsic pathway. Dose-dependent cleavage of procaspase-9 was observed. These results indicated that both the extrinsic and intrinsic apoptotic pathways were activated in the presence of IF. 

Mitochondria play a crucial role in the apoptotic cascade by serving as a convergent center of apoptotic signals originated from both the extrinsic and intrinsic pathways [15]. Depletion of ΔΨm causes the opening of the mitochondria permeability transition pore, which leads to the release of apoptogenic factors, such as cytochrome *c* in the apoptotic cascade, and activation of caspases [16]. Figure 5a showed that IF induced dose-dependent depletion of ΔΨm in CNE cells while Figure 5b showed a dose-dependent increase of cytochrome *c* in the cytosol in the presence of IF.

Tightly mediating the mitochondrial apoptotic pathway are three groups of Bcl-2 family proteins, namely, (i) Bcl-2-like survival factors which act as anti-apoptotic proteins including Bcl-2 and Bcl-xL; (ii) Bax-like death factors which serve as pro-apoptotic proteins including Bax and finally (iii) BH3-only death factors which are pro-apoptotic protein, such as Bad and Bim [17]. In response to an apoptotic stress, the BH3-only proteins were activated and interacted with Bcl-2-like survival factors on the outer mitochondrial membrane. Such interaction triggered the release of Bax-like pro-apoptotic factors which underwent a conformational change and inserted themselves into the outer mitochondrial membrane. Such insertion induced membrane permeabilization and the release of caspase-activating and other pro-apoptotic factors [18]. From the net ratio between the pro- and anti- apoptotic proteins, cells undergone apoptosis can be deduced. Our results showed that IF increased the expression of pro-apoptotic proteins, including Bax, Bad, Bim, Bik and Bmf, but decreased the expression of anti-apoptotic proteins such as Bcl-2, Bcl-xL and Mcl-1 when its concentration levels increased (Figure 6c). These observations implied that the net ratio of the two types of proteins shifted in favor of apoptosis. It was reported that Bcl-2 was detected in most samples (80%) of undifferentiated nasopharyngeal carcinoma NPC [19]. Also, the expression of Bcl-2 protein was significantly higher in NPC tissues than in both the normal noncancerous nasopharyngeal epithelia (NPE) and hyperplasic NPE [20]. However, significant down-regulation of Bcl-2 in CNE cells in the presence of IF was observed in our investigation which suggested that IF was effective in controlling the NPC. 

## 3. Conclusions

In conclusion, the phytochemical in *C. minima* that possessed strong antiproliferative effect on the human nasopharyngeal carcinoma CNE cells is 2β-(isobutyryloxy)florilenalin (IF), a sesquiterpene lactone. Both extrinsic and intrinsic apoptotic pathways were involved in its action. In the extrinsic pathway, IF activated caspase-8, which further induced the activation of caspase-3 and -7. In the intrinsic pathway, IF regulated the levels of Bcl-2 family proteins, followed by depletion of mitochondrial membrane potential (ΔΨm), the release of cytochrome *c* to the cytosol, the activation of caspase-9 and other downstream caspases, and finally the induction of apoptosis. Such activities as a result of the presence of IF may imply that *C. minima* deserves more extensive investigation for its potential medicinal application in the treatment of NPC and other cancers.

## 4. Experimental

### 4.1. General

MTT dye [3-(4,5-dimethylthiazol-2-yl)-2,5-diphenyl tetrazolium bromide)], RPMI-1640 medium, propidium iodide (PI), 4',6-diamidino-2-phenylindole (DAPI) and 5,5’,6,6’-tetrachloro-1,1’,3,3’-tetraethylbenzimidazolcarbocyanine iodide (JC-1) were purchased from Sigma (St. Louis, MO, USA); DMEM medium and fetal bovine serum (FBS) were obtained from Gibco (Rockville, MD, USA); ELISA-BrdU chemiluminescence assay kit, cytotoxicity detection kit and complete™ protease inhibitor cocktail were purchased from Roche (Germany); Bradford reagent were purchased from Bio-Rad (Hercules, CA, USA); Caspase-3 substrate (Ac-Asp-Glu-Val-Asp-*p*NA) was purchased from Calbiochem (Germany); Anticaspase-3, anticaspase-7, anticaspse-8, anticaspase-9, anti-PARP [poly(ADP-ribose) polymerase], anti-Bax, anti-Bad, anti-Bim, anti-Bik, anti-Bmf, anti-Bcl-xL, anti-Mcl-1, antirabbit secondary horseradish peroxidase antibody, and chemiluminescent substrate were purchased from Cell Signal Technology (Beverly, MA); Anti-Bcl-2, anti-β-Actin and antimouse secondary horseradish peroxidase antibody were obtained from Santa Cruz Biotech (Santa Cruz, CA, USA); Anti-cytochrome *c* was obtained from BD Biosciences (San Jose, CA, USA). All other chemicals were reagent grade.

Mass spectroscopy was measured by a gas chromatography-mass spectrometry (GC-MS) equipment (Agilent Technologies 6890/5973N, Palo Alto, CA, USA). An HP-5 ms capillary column (30 m × 0.25 mm, i.d 25 µm film thickness) was used. Helium was used as the carrier gas at a constant flow rate of 1.5 mL/min. The injected sample volume was 1 µL. The oven temperature was kept at 60 °C and programmed to 150 °C at the rate of 3 °C/min and kept constant for 5 min. Then the temperature was programmed to 240 °C at the rate of 1.5 °C/min and kept constant for 10 min. The final temperature was increased to 300 °C at the rate of 2 °C /min, and kept constant for 10 min. MS were recorded at 70 eV. The ^1^H- (400 MHz) and ^13^C-NMR (100 MHz) spectra were recorded in CDCl_3_ on a Bruker AVANCE 400 spectrometer (Bruker, Germany). 

### 4.2. Extraction, fractionation and isolation guided by in vitro cell viability assay

The aerial part of *C. minima* was collected in Baiyun mountain (Guangzhou, People’s Republic of China). A voucher specimen (06115202) has been deposited in the Department of Biology, the Chinese University of Hong Kong. Since most medicinal plants are traditionally administered as aqueous decoctions, an aqueous extract of *C. minima* was prepared to evaluate its antiproliferation activity. Briefly, the dried herb (10 g) was decocted twice with distilled water (100 mL) for 2 h. The decoction was filtered, the filtrates were combined, and lyophilized to the dried aqueous extract (1.56 g). An ethanolic extract of *C. minima* was also prepared. Briefly, the herb (2.2 kg) was macerated 4 times successively in 95% ethanol (5 L) at 20 °C for 4 days. The supernatants were filtered, concentrated *in vacuo*, and lyophilized into the dried ethanolic extract (397 g). In the preliminary test, the ethanolic extract was found to be more effective on the CNE cells than the aqueous one. Thus, the ethanolic extract was subjected to further fractionation with organic solvents. Briefly, the ethanolic extract (300 g) was suspended in distilled water (2 L) and partitioned sequentially with hexane (2 L × 5), EtOAc (2 L × 5) and *n*-butanol (2 L × 5). The solvent fractions were concentrated *in vacuo*. The remaining residue was lyophilized. The hexane fraction, which was found to be the most potent among other fractions, was subjected to further isolation. Briefly, the hexane fraction (50 g) was chromatographed on a silica gel column (70-230 mesh, 1.2 kg, Merck; diameter: 7 cm, length: 100 cm) by eluting it with a hexane-EtOAc gradient system (100:0, 99:1, 98:2, 96:4, 94:6, 92:8, 88:12, 80:20 and 0:100, [v/v]; each step used 1 L of solvent). Eleven fractions were produced on the basis of the TLC profiles. Fraction 11 (hexane-EtOAc, 0:100, 15 g) was rechromatographed on a silica gel column (70-230 mesh, 750 g, Merck; diameter: 5 cm, length: 60 cm) with a gradient of hexane-EtOAc (92:8, 90:10, 0:100, [v/v]; each step used 2.5 L of solvent) to yield nine sub-fractions. Sub-fraction 9 (hexane-EtOAc, 0:100, 8 g) was further purified by repeated column chromatographies on a sephadex LH-20 column (25-100 μm, Fluka, Switzerland; diameter: 1.5 cm, length: 30 cm) using a mixture of chloroform-methanol (5:5, v/v) to yield *2β-(isobutyryloxy)florilenalin* (IF, 50 mg, Figure 1). Colorless gum; EI-MS *m*/*z*: 334 [M]^+^; ^1^H-NMR: *δ* 1.13 (3H, d, *J* = 0.8 Hz, H-18), 1.14 (3H, d, *J* = 0.7 Hz, H-19), 1.23 (3H, s, H-15), 1.60 (1H, m, H-6β), 1.86 (1H, br d, *J* = 15.7 Hz, H-3β), 2.10 (1H, dd, *J* = 4.7, 14.2 Hz, H-6α), 2.17 (1H, s, H-5), 2.17 (1H, s, H-1), 2.20 (1H, dd, *J* = 5.8, 15.7 Hz, H-3α), 2.30 (1H, dd, *J* = 11.7, 12.4 Hz, H-9β), 2.52 (1H, m, H-17), 2.70 (1H, dd, *J* = 3.6, 12.4 Hz, H-9α), 3.23 (1H, m, H-7), 4.59 (1H, m, H-8), 4.85 (1H, s, H-14’), 5.04 (1H, s, H-14), 5.27 (1H, dd, *J* = 2.4, 5.2 Hz, H-2), 5.65 (1H, d, *J* = 2.4 Hz, H-13’), 6.27 (1H, d, *J* = 2.8 Hz, H-13); ^13^C-NMR: *δ* 18.7 (-CH_3_), 18.8 (-CH_3_), 25.3 (C-15), 29.7 (C-6), 34.2 (C-17), 39.6 (C-9), 42.2 (C-7), 49.1 (C-3), 51.6 (C-5), 53.4 (C-1), 73.5 (C-2), 78.4 (C-4), 80.4 (C-8), 115.6 (C-14), 122.6 (C-13), 139.2 (C-11), 139.8 (C-10), 169.7 (C-12) and 176.4 (C-16). 

### 4.3. Cell culture

Human nasopharyngeal cancer cells (CNE) and human normal skin cells (Hs68) were purchased from the Cell Bank of Type Culture Collection of Chinese Academy of Sciences (Shanghai, People’s Republic of China) and the American Type Culture Collection (Rockville, MD, USA), respectively. CNE cells were maintained in RPMI-1640 medium, while Hs68 cells were maintained in DMEM medium. Both media were supplemented with 10% fetal bovine serum and 1% penicillin-streptomycin at 37 °C in a humidified incubator with 5% CO_2 _atmosphere. 

### 4.4. Cell viability analysis by MTT assay

Cell viability was determined by a MTT assay. Briefly, different concentrations of samples were added to the cells after 24-h incubation in a 96-well microtiter plate. Following incubation for specific times, MTT solution [20 μL, 5 mg/mL in phosphate buffered saline (PBS)] was added to each well, and the cells were further incubated for 4 h. Excess medium was removed and replaced by DMSO (150 μL) to dissolve the formazan crystals. The optical densities were determined by a microplate spectrophotometer (SPECTRAmax 250, Molecular Devices, MN, USA) at the wavelength of 570 nm.

### 4.5. Cell proliferation analysis by BrdU assay

Cell proliferation was determined by the BrdU (5-bromo-2’-deoxyuridine) chemiluminescence assay which was based on the measurement of BrdU incorporation during DNA synthesis in proliferating cells. The ELISA-BrdU chemiluminescence assay kit (Roche, Germany) was employed. Briefly, after incubating the cells with various concentrations of IF for 24 h in a 96-well microtiter plate, BrdU labeling solution (100 μM, final concentration) was added, and the plate was incubated for 2 h. The labeling solution was removed and the cells were fixed with FixDenat solution for 30 min. After removal of the FixDenat solution, anti-BrdU-POD solution was added and further incubated for 1.5 h. The chemiluminescence intensity was determined according to the manufacturer’s instruction. Cell proliferation was expressed as the percentage relative to the control which contained no IF.

### 4.6. Cytotoxicity determination by lactate dehydrogenase (LDH) assay

Cytotoxicity as reflected by the LDH activity was determined by the Cytotoxicity Detection Kit (Roche, Germany). Briefly, both treated and untreated cells were centrifuged at 500 *g* for 10 min at 20 °C. Supernatants (100 μL) were collected and transferred to a new 96-well microtiter plate. LDH activity was determined according to the manufacturer’s instruction. Cytotoxicity was evaluated by measuring the percentage of LDH released into the medium. Cells treated with 1% Triton X-100 were used as positive control (i.e. 100% lysis of the cells).

### 4.7. DNA contents analysis by flow cytometry

The effect of IF on the cell cycle distribution of CNE cells were determined by flow cytometric analysis. Briefly, treated or untreated CNE cells were harvested and washed with PBS twice. Cells were stained with freshly prepared DNA staining solution containing propidium iodide (PI, 20 μg/mL), RNase A (200 μg/mL) and 0.1% Triton X-100 after fixed in 70% ethanol at -20 °C overnight. Stained cells were then subjected to analyze by a flow cytometer (EPICS XL Flow cytometry, Beckman Coulter, Miami, FL, USA). Cells displaying hypodiploid DNA content were quantified and regarded as the apoptotic population. In each experiment, 10,000 events per sample were recorded.

### 4.8. Nuclear staining with DAPI

Morphological changes in the nuclear chromatin of cells undergoing apoptosis were revealed by a nuclear fluorescent dye, DAPI. Briefly, cells after treatment for 72 h were harvested, washed with PBS twice. After fixed with 4% paraformaldehyde and permeated with 0.1% Triton X-100, the cells were stained with 2 μg/mL of DAPI for 15 min. The cells were then observed under a fluorescence microscope (Nikon Eclipse 80i, Japan).

### 4.9. Mitochondrial membrane potential (ΔΨm) assay

A cationic dye JC-1 was used to measure the depletion of mitochondrial membrane potential. JC-1 exhibits potential-dependent accumulation in mitochondria, indicated by a fluorescence emission shift from green to red. Briefly, after treatment with IF for 72 h, cells were washed with PBS twice and stained with 10 μM of JC-1. After incubation in dark at 37 °C for 15 min, cells were analyzed by the flow cytometer. Data are expressed in percentage of cells with changed ΔΨm. 

### 4.10. Caspase activity assay

Caspase activity was determined by using the colorimetric peptite caspase-3 substrate Ac-Asp-Glu-Val-Asp-*p*NA. Briefly, cell lysates were placed in 96-well plate, and 100 μM of the substrate was added. The plate was incubated at 37 °C for 1 h and caspase activity was determined by the microplate spectrophotometer at the wavelength of 405 nm.

### 4.11. Western blot analysis

To obtain cytosolic proteins, cells were harvested and lysed in hypotonic lysis buffer (250 mM sucrose, 20 mM Hepes, 1.5 mM MgCl_2_, 10 mM KCl, 1 mM EGTA, 1 mM DTT, complete™ protease inhibitor cocktail) for 30 min on ice. Then the cells were homogenized in a Dounce homogenizer with optimal gentle strokes. After centrifugation at 1,000 *g* for 10 min at 4 °C to remove the unlysed cells and nuclei, the supernatants were further centrifuged at 14,000 *g* for 15 min at 4 °C. The supernatant was collected as the cytosolic proteins. The total cellular proteins were extracted by lysing cells in a lysis buffer [1% IGEPAL CA 630 (Fluka, Switzerland), 150 mM NaCl, 50 mM Tris-HCl (pH 7.5), complete™ protease inhibitor cocktail] for 30 min on ice. Lysates were clarified by centrifugation for 15 min at 14,000 *g*. The supernatant was collected as the total cellular proteins. Protein concentrations were determined with Bradford reagent. Equal amounts of proteins (20-40 μg protein) were resolved on 7.5-12% SDS-polyacrylamide gels, followed by electrophoretic transfer to PVDF (Polyvinylidene fluoride, Millipore, Bedford, MA, USA) membranes. The membranes were blocked with 5% non-fat dry milk in TBST (Tris-Buffered Saline Tween-20) for 1 h, followed by incubation sequentially with primary antibodies at 4 °C and the horseradish peroxidase-conjugated secondary antibodies at 20 °C. Protein bands were detected on X-ray film (Fuji, Japan) using the chemiluminescent substrate. 

### 4.12. Statistical analysis

All results were expressed as mean ± SD from three independent experiments. Statistical analysis was performed using a two-tailed Student’s *t*-test. Difference with *p* < 0.05 (*) was considered statistically significant.
